# Antibody responses to *P. falciparum* blood stage antigens and incidence of clinical malaria in children living in endemic area in Burkina Faso

**DOI:** 10.1186/s13104-017-2772-9

**Published:** 2017-09-08

**Authors:** Mariama K. Cherif, Oumarou Ouédraogo, Guillaume S. Sanou, Amidou Diarra, Alphonse Ouédraogo, Alfred Tiono, David R. Cavanagh, Theisen Michael, Amadou T. Konaté, Nora L. Watson, Megan Sanza, Tina J. T. Dube, Sodiomon B. Sirima, Issa Nebié

**Affiliations:** 1 Centre National de Recherche et de Formation sur le Paludisme, Ouagadougou, Burkina Faso; 2 Université Polytechnique de Bobo-Dioulasso, Bobo-Dioulasso, Burkina Faso; 3 Université de Ouagadougou, Ouagadougou, Burkina Faso; 4 Institute of Immunology and Infection Research, University of Edinburgh, Scotland, UK; 5 Department of Clinical Biochemistry Statens Serum, Copenhagen, Denmark; 6 The EMMES Corporation, Rockville, MD USA

**Keywords:** *P. falciparum* infection, Malaria transmission sessions, Age, Antigens

## Abstract

**Background:**

High parasite-specific antibody levels are generally associated with low susceptibility to *Plasmodium falciparum* malaria. This has been supported by several studies in which clinical malaria cases of *P. falciparum* malaria were reported to be associated with low antibody avidities. This study was conducted to evaluate the role of age, malaria transmission intensity and incidence of clinical malaria in the induction of protective humoral immune response against *P. falciparum* malaria in children living in Burkina Faso.

**Methods:**

We combined levels of IgG and IgG subclasses responses to *P. falciparum* antigens: Merozoite Surface Protein 3 (MSP3), Merozoite Surface Protein 2a (MSP2a), Merozoite Surface Protein 2b (MSP2b), Glutamate Rich Protein R0 (GLURP R0) and Glutamate Rich Protein R2 (GLURP R2) in plasma samples from 325 children under five (05) years with age, malaria transmission season and malaria incidence.

**Results:**

We notice higher prevalence of *P. falciparum* infection in low transmission season compared to high malaria transmission season. While, parasite density was lower in low transmission than high transmission season. IgG against all antigens investigated increased with age. High levels of IgG and IgG subclasses to all tested antigens except for GLURP R2 were associated with the intensity of malaria transmission. IgG to MSP3, MSP2b, GLURP R2 and GLURP R0 were associated with low incidence of malaria. All IgG subclasses were associated with low incidence of *P. falciparum* malaria, but these associations were stronger for cytophilic IgGs.

**Conclusions:**

On the basis of the data presented in this study, we conclude that the induction of humoral immune response to tested malaria antigens is related to age, transmission season level and incidence of clinical malaria.

## Background

Children and pregnant women in sub-Saharan African are carrying the global *P. falciparum* malaria burden. It has been shown that there is a relationship between antibody production (levels, isotype, or function) and susceptibility to clinical malaria [1, 2]. Bouharoun-Tayoun and Druilhe found a significance difference in the distribution of immunoglobulin (Ig) subclasses between clinically protected and non-protected individuals. In their study, cytophilic isotypes (IgG1 and IgG3) are being reported to be dominant in protected individuals compared to non-protected individual [3]. Similar findings were observed also for MSP2, MSP3 and GLURP where cytophilic antibodies were predominant in protected individuals, while non cytophilic antibodies were predominant in non protected individuals [4–7].

There are several merozoite surface proteins with no specific function, including MSP2; MSP3 and GLURP [8]. Many of these antigens have been evaluated or developed as potential vaccine antigens [9]. Immuno-epidemiological studies have shown that IgG to these antigens is associated with lower parasite density [10, 11] and the absence of disease [12] in children living in West-Africa, and that levels of IgG responses to these antigens increased quickly with age and with level of exposure [6, 13, 14].

MSP3 and GLURP have been tested in malaria vaccine antigens that have already undergone phase 1 trials. The efficacies of these malaria vaccines will be tested in phase II and III vaccine trials in Africa. For the purpose of the evaluation of the efficacy of these planned vaccine trials, it is important to investigate the induction of humoral immune response in protection against these vaccines antigens. However, the epidemiological evidence of the protective effect of naturally acquired anti-merozoite responses is not specifique. There could be many reasons for these inconsistencies. In malaria endemic areas the rate at which natural immunity develops is dependent on age, intensity and stability of exposure to *P. falciparum*, endemicity of malaria and clinical incidence [15, 16]. This study was conducted to evaluate the role of age, malaria transmission intensity and incidence of clinical malaria in the induction of protective humoral immune response against malaria vaccine candidates prior to clinical trials in children living in Burkina Faso.

## Methods

### Study area

The study was conducted in four villages randomly selected out of the 74 villages of the Saponé Health District (SHD). The SHD is located at the center of Burkina Faso at 50 km south-west of Ouagadougou. In SHD, the climate is characteristic of the Sudanese savannah with two seasons: a rainy season from June to October and a dry season from November to May where the malaria transmission is nearly absent. Malaria is endemic and its transmission in these areas is very high during the rainy season. *P. falciparum* is the deadliest specie that causes more than 95% of infections [17]. The principal vectors are *An. gambiae*, and *An. funestus*. From February to May, the number of infectious bites per person per night (Entomological Inoculation Rate (EIR)) was negligible. However, the EIR increased from June to September and decreased from September to November and remained low until the next rainy season [6, 17].

### Study population

Study population was children from 6 months to 5 years fulfilling the following inclusion and exclusion criteria.

Inclusion criteria were (i) written/thumb printed informed consent obtained from the parent or legal guardian of each child enrolled to the study; (ii) permanent resident in the study area for at least 3 months prior to enrollment and expected to remain at least for the all the period of longitudinal follow up; (iii) aged between 6 months to 5 years.

Exclusion criteria were (i) major congenital defect or any chronic disease (including immune, cardiovascular, hepatic, HIV and renal) diagnosed by a physician/nurse based on medical history and physical examination; (ii) anemia defined as hemoglobin value less than 6 g/dl and (iii) any other circumstances and condition suspected by the physician to be the risk for the child health.

Only children fulfilling study inclusion criteria and attending both cross-sectional surveys were enrolled to the study. Data obtained by cross-sectional survey was used to reach study objectives. Children were enrolled for the assessment of malaria infection and immunological endpoints in relation to protection against clinical malaria, age and malaria transmission level.

### Sample collection

Two cross-sectional surveys were carried out during low (January 2007) and high (September 2007) malaria transmissions seasons. During each cross-sectional survey, 5 ml of venous blood in a tube containing EDTA was collected from each child for a complete blood count. The remaining blood was centrifuged and aliquots of plasma were created and stored at −20 °C for immunological analysis. Thick and thin blood films were prepared from finger prick for microscopy diagnosis of malaria. Axillary temperature was measured at once. Children with fever, defined as axillary temperature ≥37.5 °C or history of fever reported within the last 24 h, had a malaria rapid diagnostic test (RDT) performed. A child with a positive test result was referred to the nearest health center for appropriate treatment of malaria which was given free of charge.

A longitudinal survey with active case detection of malaria episodes was conducted starting from the beginning of the first cross-sectional survey. Twice weekly, each child was monitored clinically by a study nurse who resided in the village. During each visit, information regarding health status was recorded and axillary temperature was measured as described previously. If the child had fever or history of fever reported within the last 24 h, a RDT was performed. In addition, thick and thin blood films were prepared and sent to the Centre National de Récherche et Formation sur le Paludisme (CNRFP) laboratory for the determination of parasite density by using light microscopy. Patients with positive RDTs were referred at once to the nearest health care center for free treatment.

### Malaria infection diagnosis

*Plasmodium falciparum* parasite is identified by examining thick and thin blood films. Each slide was air-dried and stained with 5% Giemsa to give a parasites a distinctive appearance and was read by two different laboratory technicians. A slide was declared negative only after reading against 2000 white blood cells without observation of a malaria parasite. For blood smears collected during the cross-sectional survey, the number of parasites per microliter of blood was calculated according to the number of white blood cells obtained from a completed blood count; however, parasite densities for slides collected during the longitudinal survey were calculated assuming an average of 8000 white blood cells/µL of blood. In case of difference over the presence or absence of malaria parasites between different readers, or if parasite density estimates differed by more than 30%, the slide was read one again by a third laboratory technician. The arithmetic mean of the two final readings was used as the final parasite density. If there was no agreement after the third reading, the arithmetic mean of the two most closed results was considered.

### Immunological analysis

IgG and IgG subclass levels to merozoite surface antigens MSP3, MSP2a, MSP2b, GLURP R0 and GLURP R2 (Table 1) were measured according to Afro Immuno Assay 2 (AIA2), standard operating procedure (SOP Number: AIA-001-02) [6, 14]. In brief, microtiter plates (Maxisorp Nunc –F 96 442404, Denmark) were coated with the appropriate antigens at 0.5 µg/ml and were incubated at 4 °C overnight. The plates were then; blocked with PBS Tween 20 (PBS with 5% milk powder, 0.1% Tween-20) for 1 h. Plasma samples diluted 1:200 in serum dilution buffer (PBS with 2.5% milk powder, 0.1% Tween-20 and 0.02% Na-azide) were added in duplicate and incubated at room temperature for 2 h. Plate were washed four times between each step with washing buffer (PBS with 0.1% Tween-20 and 0.5 M NaCl). 50 μL per well of respective conjugated anti-human antibodies were added. Antibodies used were peroxidase-conjugated goat anti-human IgG (γ) (H10007) (Invitrogen Corporation, CA, USA) (1:80,000) and the IgG subclasses antibodies (The Binding Site Group Ltd, UK): peroxidase-conjugated sheep anti-human IgG1 (AP006) (1:5000), peroxidase-conjugated sheep anti-human IgG2 (AP007) (1:2000), peroxidase-conjugated sheep anti-human IgG3 (AP008) (1:10,000) and peroxidase-conjugated sheep anti-human IgG4 (AP009) (1:1000), all diluted in dilution buffer (0.1% Tween20 + 2.5% milk powder in PBS) and incubated for 1 h at room temperature. After washing, the plates were developed with TMB (3,3′,5,5′-Tetramethylbenzidine) from Taastrup, Denmark (Kem-En-Tec Diagnosis A/S, Taastrup, Denmark) substrate and reactions stopped after 30 min by adding 50 µL of 0.2 M of sulfuric acid per well. Antibody levels, measured as optical density (OD) were determined at 450 nm with a reference at 630 nm, using a Biotek ELx808 microplate reader (Winooski, Vermont 05404-0998 USA). The OD values of the test samples were converted into Arbitrary Units (AU) ADAMSEL b040, Ed Remark© 2009) by means of interpolation from a standard curve on each plate, obtained by using 12 serial dilutions of a pool of positive hyperimmune sera (from CNRFP site). Positive control plasmas were obtained from positive Burkinabè adults over 20 years old, living in malaria hyper-endemic areas and negative controls were Danish (never exposed to malaria) plasma samples from Statens Serum Institute (Copenhagen, Denmark). Samples were re-tested if the coefficient of variation between duplicate absorbance values were higher than 15% and plates were also re-tested if the R2 value of the standard curve was less than 97%. A mean low cut concentration were generated for all the analysis at 0.0028 AU.

**Table 1:** Characteristics of antigens used [8, 14, 19, 20]

Merozoite surface antigens			
Antigen	Synthesis stage	Allelic type	Polypeptides
MSP3	Merozoite surface		
MSP2	Merozoite surface	A	1–184 of strain CH150/9
		B	22–247 of strain Dd_2_
GLURP	All stages of parasite	R0	N-terminal non repetitive region GLURP_94–489_
		R2	C-terminal repetitive region GLURP_705–1178_

### Data analysis

Data was performed using EPI info version 6.0. Data generated from assays in the form of ELISA OD values were entered into Microsoft Excel worksheets. SAS software Version 9.2 was used to perform analyses.

Incidence rates of malaria which included varied thresholds of parasitemia (parasitemia > 0, parasitemia ≥ 2500, and parasitemia ≥ 5000 asexual forms/µL of blood) and axillary temperature ≥37.5 °C or history of fever within 24 h were calculated for the following time periods: date of 1st cross sectional survey to date of 2nd cross sectional survey. Malaria episodes occurring within 28 days of a previous episode were not treated as incident episodes. When calculating person time in analyses of multiple malaria episodes, 28 days following a new episode was subtracted from total time at risk. This was done to ensure that the infection causing the episode was recorded and subsequent episodes within the 28-day window were not counted. Overall incidence of malaria was calculated using the number of malaria events divided by time at risk. Geometric means for antigens were estimated using log_2_ transformed values. The Wilcoxon signed-rank sum test was used to compare geometric mean parasite densities across transmission seasons. Age adjusted incidence rate ratios against multiple malaria episodes was determined by Poisson regression.

## Results

### Characteristics of study population

We performed immunological analysis of samples from 325 children under 5 years old (mean age 2.71 years and the sex ratio M/F 1.12). Participants were followed up from February 2007 (low malaria transmission season) to December 2007 (peak of high malaria transmission season) to assess malaria clinical episodes and incidence.

As shown in Table 2, the prevalence of *P. falciparum* infection during low and high transmission seasons was 59.69 and 50.76%, respectively. By contrast, the mean parasite density was lower during low malaria transmission season compared to high transmission season; 2627 parasites/µL versus 6042 parasites/µL. Using increasingly stringent definitions of malaria by increasing the parasitemia threshold, during each of low and high transmission seasons, the incidence of malaria decreased. However, regardless of the clinical malaria definition, the incidence rates were always higher during high malaria transmission season.

**Table 2:** Study population characteristics

Characteristics	Number of children	Low malaria transmission season	High malaria transmission season
Baseline *P. falciparum*			
Negative	325	126 (43.3%)	160 (49.2%)
Positive	325	194 (59.7%)	165 (50.8%)
Geometric means of parasites density/μL	325	2627	6042
Incidence of malaria (95% CI)			
>0	325	2.4 (2.2, 2.6)	2.9 (2.6, 3.3)
≥2500	325	1.7 (1.5, 1.9)	2.3 (2.0, 2.6)
≥5000	325	1.5 (1.3, 1.7)	2.0 (1.8, 2.3)

### Relationship between IgG levels and age

**Fig. 1:**
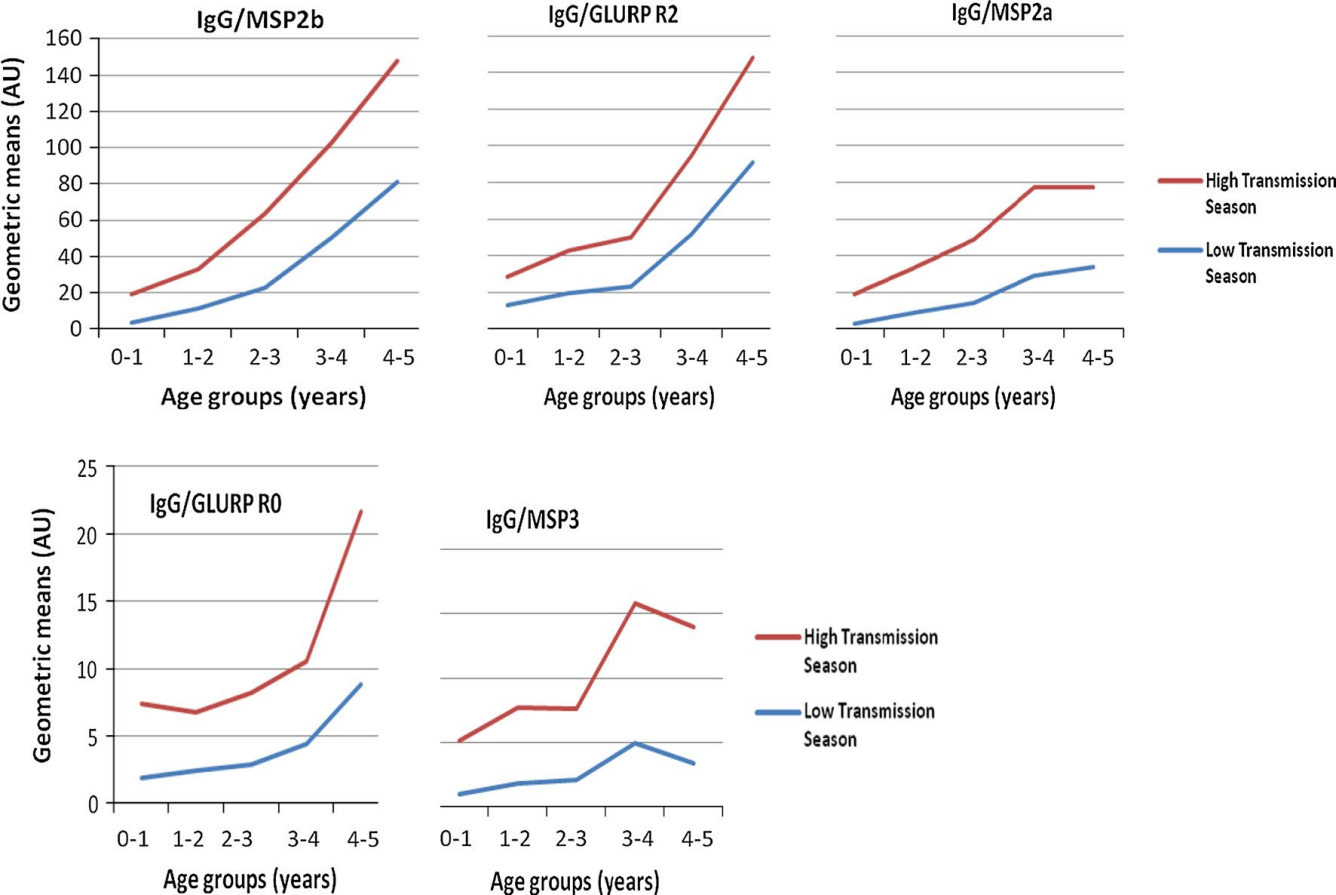
IgG level in children during both malaria transmission seasons by age groups: MSP2b, Merozoite Surface Protein 2b; GLURP R2, Glutamate Rich Protein R2; MSP2a, Merozoite Surface Protein 2a; GLURP R0, Glutamate Rich Protein R0; MSP3, Merozoite Surface Protein 3

Children were categorized into five age groups to assess the age-dependent response of IgG to the tested antigens (MSP3, MSP2a, MSP2b, GLURP R0 and GLURP R2). In our study, older children had higher IgG responses than younger children for all tested antigens. Results are shown in Fig. 1. The difference was statistically different or borderline between the defined age groups in both transmission seasons: MSP3 (p < 0.0001 and p = 0.0069), MSP2a (p < 0.0001 and p = 0.0037), MSP2b (p < 0.0001 and p < 0.0001), GLURP R0 (p < 0.0001 and p = 0.0508) and GLURP R2 (p < 0.0001 and p = 0.0010) respectively during low and high malaria transmission seasons.

### Relationship between antibody levels and malaria transmission seasons

Levels of specific IgG and IgG subclasses antibodies in 325 children were compared between low and high malaria transmission seasons (Table 3). Overall IgG levels to all antigens except for GLURP R2 were significantly higher during high malaria transmission season compared to the measurement done during low transmission season, MSP3 (p < 0.001), MSP2a (p < 0.001) and GLURP R0 (p < 0.001). The reverse effect was observed with GLURP R2 for which, the level of specific IgG was higher during low malaria transmission season (p = 0.0372).

**Table 3:** IgG and IgG subclass levels in children during both periods of the malaria transmission seasons

Antigen	Isotype	N	Low transmission season		High transmission season		p*
			Geometric mean	(95% CI)	Geometric mean	(95% CI)	
MSP3	IgG	325	2.55	(2.12, 3.08)	7.29	(6.12, 8.67)	<.0001
	IgG1	325	4.35	(3.85, 4.92)	9.41	(8.47, 10.44)	<.0001
	IgG2	325	4.95	(4.48, 5.47)	4.96	(4.53, 5.42)	0.065
	IgG3	325	1.33	(1.08, 1.63)	2.89	(2.42, 3.45)	<.0001
	IgG4	325	3.15	(2.88, 3.45)	2.95	(2.64, 3.29)	<.0001
MSP2a	IgG	325	14.82	(11.92, 18.43)	33.21	(27.79, 39.67)	<.0001
	IgG1	325	3.60	(3.11, 4.16)	5.73	(4.92, 6.67)	0.0012
	IgG2	325	3.38	(3.08, 3.71)	3.95	(3.46, 4.51)	<.0001
	IgG3	325	12.43	(10.22, 15.11)	15.04	(12.48, 18.13)	0.0147
	IgG4	325	2.52	(2.08, 3.05)	2.64	(2.21, 3.15)	0.0539
MSP2b	IgG	325	24.50	(19.63, 30.57)	37.01	(31.38, 43.66)	0.3356
	IgG1	325	5.66	(4.89, 6.55)	13.15	(11.58, 14.93)	<.0001
	IgG2	325	4.29	(3.94, 4.66)	3.62	(3.12, 4.21)	0.1138
	IgG3	325	11.90	(9.56, 14.81)	13.73	(11.19, 16.85)	0.4537
	IgG4	325	1.65	(1.31, 2.08)	1.99	(1.64, 2.42)	0.3316
GLURP-R0	IgG	325	3.65	(3.03, 4.41)	6.31	(5.08, 7.84)	<.0001
	IgG1	325	3.76	(3.24, 4.35)	3.38	(2.87, 3.98)	0.2451
	IgG2	325	1.13	(1.01, 1.26)	4.45	(3.93, 5.04)	<.0001
	IgG3	325	1.46	(1.24, 1.71)	0.97	(0.82, 1.14)	<.0001
	IgG4	325	1.62	(1.42, 1.85)	0.34	(0.30, 0.38)	<.0001
GLURP-R2	IgG	325	33.45	(27.73, 40.36)	31.60	(26.44, 37.77)	0.0372
	IgG1	325	17.59	(14.02, 22.06)	41.35	(34.08, 50.17)	<.0001
	IgG2	325	7.42	(5.84, 9.43)	30.11	(26.56, 34.14)	<.0001
	IgG3	325	16.77	(13.17, 21.36)	12.87	(10.02, 16.52)	0.0001
	IgG4	325	6.87	(6.01, 7.85)	5.07	(4.40, 5.85)	<.0001

*  p ≤ 0.05 was considered as statistically significant

IgG isotype levels were always higher in children during high malaria transmission period compared to low transmission period but the difference were not significant for IgG2 to MSP3 (p = 0.0738), IgG4 to MSP2a, IgG2, IgG3 and IgG4 to MSP2b (p = 0.10, p = 0.39 and p = 0.35 respectively) and IgG1 to GLURP R0 (p = 0.2085).

### Effect of clinical malaria incidence on antibody levels

Clinical malaria incidence rates was performed as previously described [6, 14, 17] (Tables 4, 5, 6). In a 1-year follow-up, it appears that IgG responses to GLURP R0 and R2 were statistically associated with reduced risk of clinical malaria when adjusted for age. Higher levels of IgG to MSP2b were associated with a low risk of clinical malaria, the parasite threshold were brought to 2500 and 5000 asexual stages/µL of blood (2500: p = 0.03; 5000: p = 0.02). There was no evidence of such associations for total IgG to MSP3 and MSP2a (Table 4).

**Table 4:** Age adjusted incidence rate ratios (IRR) (95% CI) for the association between total IgG from low to the end at high malaria transmission season

Antigen	Parasitemia > 0		Parasitemia > 2500		Parasitemia > 5000	
	Age adjusted (95% CI)	p	Age adjusted IRR (95% CI)	p	Age adjusted IRR (95% CI)	p*
MSP3	0.98 (0.947, 1.008)	0.14	0.97 (0.934, 1.012)	0.2	0.97 (0.932, 1.013)	0.17
MSP2a	0.99 (0.968, 1.021)	0.66	0.98 (0.949, 1.016)	0.29	0.98 (0.944, 1.012)	0.20
MSP2b	0.99 (0.963, 1.019)	0.52	0.96 (0.928, 0.996)	0.031	0.96 (0.924, 0.994)	0.02
GLURP-R0	0.96 (0.927, 0.987)	0.006	0.94 (0.902, 0.977)	0.002	0.93 (0.891, 0.969	0.0006
GLURP-R2	0.94 (0.907, 0.968)	0.0001	0.91 (0.875, 0.952)	0.00002	0.91 (0.873, 0.953)	0.00003

*  p ≤ 0.05 was considered as statistically significant

**Table 5:** Age-adjusted Incidence rate ratios for cytophilic IgG with malaria incidence from low to the end of high transmission season

Antibodies/antigen		Parasitemia > 0		Parasitemia > 2500		Parasitemia > 5000	
		Age adjusted (95% CI)	p	Age adjusted (95% CI)	p	Age adjusted (95% CI)	p*
IgG1	MSP3	0.94 (0.897, 0.989)	0.016	0.93 (0.872, 0.988)	0.02	0.93 (0.874, 0.995)	0.034
	MSP2a	0.99 (0.956, 1.031)	0.7	0.98 (0.936, 1.029)	0.44	0.97 (0.919, 1.014)	0.16
	MSP2b	1.00 (0.959, 1.037)	0.90	0.98 (0.936, 1.033)	0.5	0.98 (0.932, 1.032)	0.45
	GLURP-R0	0.93 (0.892, 0.965)	0.0002	0.90 (0.851, 0.942)	0.00002	0.89 (0.845, 0.939)	0.00002
	GLURP-R2	0.96 (0.932, 0.982)	0.001	0.94 (0.914, 0.976)	0.0007	0.94 (0.912, 0.977)	0.001
IgG3	MSP3	0.97 (0.940, 0.996)	0.024	0.95 (0.913, 0.983)	0.004	0.94 (0.908, 0.981)	0.003
	MSP2a	0.99 (0.960, 1.020)	0.49	0.97 (0.934, 1.008)	0.12	0.97 (0.929, 1.005)	0.091
	MSP2b	0.98 (0.948, 1.005)	0.1	0.95 (0.911, 0.981)	0.003	0.94 (0.903, 0.975)	0.0011
	GLURP-R0	0.98 (0.942, 1.010)	0.16	0.96 (0.918, 1.003)	0.068	0.96 (0.913, 1.001)	0.056
	GLURP-R2	0.96 (0.936, 0.982)	0.0007	0.94 (0.907, 0.966)	0.00003	0.93 (0.903, 0.964)	0.00003

*  p ≤ 0.05 was considered as statistically significant

**Table 6:** Age-adjusted Incidence rate ratios for non cytophilic IgG with malaria incidence from low to the end of high malaria transmission season

Antibodies/antigen		Parasitemia > 0		Parasitemia > 2500		Parasitemia > 5000	
		Age adjusted (95% CI)	p	Age adjusted (95% CI)	p	Age adjusted (95% CI)	p*
IgG2	MSP3	1.02 (0.963, 1.080)	0.50	1.04 (0.964, 1.113)	0.34	1.04 (0.965, 1.121)	0.3
	MSP2a	0.98 (0.923, 1.042)	0.53	0.96 (0.889, 1.033)	0.27	0.95 (0.879, 1.027)	0.2
	MSP2b	0.94 (0.877, 1.011)	0.095	0.87 (0.798, 0.950)	0.002	0.87 (0.791, 0.947)	0.002
	GLURP-R0	1.00 (0.954, 1.054)	0.92	0.99 (0.927, 1.054)	0.72	1.00 (0.935, 1.066)	0.958
	GLURP-R2	0.98 (0.959, 1.008)	0.17	0.96 (0.931, 0.992)	0.013	0.97 (0.934, 0.997)	0.034
IgG4	MSP3	0.95 (0.886, 1.014)	0.12	0.90 (0.824, 0.987)	0.024	0.90 (0.819, 0.988)	0.03
	MSP2a	0.98 (0.950, 1.011)	0.21	0.97 (0.935, 1.012)	0.16	0.97 (0.931, 1.011)	0.15
	MSP2b	0.98 (0.957, 1.007)	0.16	0.97 (0.934, 0.998)	0.038	0.96 (0.925, 0.992)	0.015
	GLURP-R0	0.98 (0.939, 1.023)	0.35	0.99 (0.941, 1.046)	0.78	0.97 (0.922, 1.030)	0.37
	GLURP-R2	0.94 (0.899, 0.990)	0.02	0.89 (0.826, 0.949)	0.0006	0.88 (0.818, 0.947)	0.0006

*  p ≤ 0.05 was considered as statistically significant

Among the four IgG isotypes, the two cytophilic classes (IgG1 and IgG3) were most associated with decreased malaria incidence. Association was strongest for IgG1 to MSP3 (p = 0.01641; p = 0.01906 and p = 0.03405), GLURP R0 (p = 0.00019; p = 0.00002 and p = 0.00002) and GLURP R2 (p = 0.00087; p = 0.00074, p = 0.00096) respectively for parasitemia threshold >0 parasites; parasitemia threshold >2500 parasites and parasitemia threshold >5000 parasites (Table 5). There was also a significant association for IgG3 to MSP3 (p = 0.02474, p = 0.00439; p = 0.00347) and GLURP R2 (p = 0.00066, p = 0.00003 and p = 0.00003).

When IgG2 and IgG4 were considered, there was no evidence of an association with malaria incidence for parasitemia >0, there was a moderate association, with borderline statistical significance of IgG4 to GLURP R2, (p = 0.01839 (Table 6). With parasitemia thresholds of >2500 parasites and >5000, the associations were significant for IgG2 to MSP2b (p = 0.00191 and p = 0.00172) and GLURP R2 (*p* = 0.01302 and p = 0.03444) and IgG4 (p = 0.02417 and p = 0.02739); MSP2b (p = 0.03784 and p = 0.015) and GLURP R2 (p = 0.00056 and p = 0.00061) respectively for parasitemia more than 2500 parasite and 5000 parasites.

## Discussion

This study aimed was to continuously support malaria vaccine development based on specific *P. falciparum* asexual stage antigens. A distinguishing characteristic of our study is the combination of the quantification of IgG and IgG subclasses to *P. falciparum* antigens (MSP3, MSP2a, MSP2b, GLURP R0 and GLURP R2) using standardized methods with age and malaria transmission season as done in previous studies [6, 14, 17]. In addition, a longitudinal survey with active case detection of malaria episodes conducted over 1 year allowed the evaluation of the parasitological status and the incidence of clinical malaria.

Our data showed that *P. falciparum* infection prevalence was high in low transmission season compared to high transmission season. This finding is surprising because most malariometric parameters have been reported to be highest during malaria high transmission seasons in seasonal and stable malaria transmission areas. However in this study there was biweekly active follow-up, where children were treated with artemisinin combination therapy (ACT) when (a) axillary temperature was ≥37.5 °C and (b) there was reported history of fever within last 24 h, with a positive rapid malaria diagnosis test. Repeated treatment with ACT during these malaria illnesses might prevent malaria infection throughout the period of greatest malarial risk. As expected, the asexual parasite load and clinical malaria incidence regardless of parasite density thresholds peaked during the malaria intense transmission period. As shown in previous studies, these patterns are characteristic of our study area [18–20].

As shown in this paper, recent studies with blood-stage antigens carried out in areas where malaria is endemic have reported increasing antibody (IgG and IgG isotypes) levels with age [6, 14]. This is consistent with the hypothesis that immunity to malaria is largely built up throughout long-term exposure to malaria parasites [21]. Increasing IgG levels with age may reflect greater cumulative exposure of older children to malaria parasites, but may also be due to older children having a more fully developed acquired immune system [22].

Seasonal variation of IgG antibody responses to malaria parasite antigens has already been assessed in malaria endemic settings, where the responses were influenced by seasonal variation [17, 23]. In our current study, the same trend was observed with the level of antibodies against asexual blood antigens (MSP3, MSP2a, MSP2b, and GLURP R0) suggesting that the acquisition of the immunity against malaria is dependent on the intensity of malaria transmission. Surprisingly the reverse observation was made for IgG to GLURP R2, showing slightly higher level of antibodies during low transmission compared with the high transmission season. As previously reported by Nebie and colleagues, it has been suggested that above certain level of transmission, antigen load may induce a disturbance of humoral response leading to lower antibody levels, which may explain this finding [17, 23]. Some evidence in support of this hypothesis was found in Madagascar, where higher quantities of circulating stable antigens were reported to down-regulate antibody responses to *P. falciparum*. However we cannot exclude that this observation may also be due to a random variation. As shown in other studies, cytophilic antibody levels to the five antigens tested in this study were higher than non-cytophilic ones independent of the malaria transmission season, emphasizing their importance in anti-malaria immunity [3, 7, 13, 21, 22, 24–26]. Many previous studies have shown that the B-cell epitopes of MSP3 and GLURP are targeted by IgG1 and IgG3, and, in conjunction with blood mononuclear cells via their FcγRII receptors, trigger the release of killing factors, such as tumor necrosis factor [1, 13, 27, 28].

Previous studies using human sera from people living in malaria endemic areas have found evidence of an association between the levels of total IgG to MSP3 and GLURP and a reduced subsequent risk of clinical malaria [29–31]. For GLURP antigens, we found that there is an association between total IgG antibodies and a lower risk of clinical malaria in the majority of the study population. While, we observed the controversial situation for MSP3. Our data show that GLURP R0 and R2-specific IgG are correlated with a reduced risk of *P. falciparum* clinical malaria, but we find no evidence for associations with MSP3, MSP2a and MSP2 for malaria episodes defined by a parasitemia threshold >0. For malaria definitions with parasitemia thresholds >2500 and >5000, in addition to GLURP R0 and GLURP R2, we observed that IgG to MSP2b was correlated with reduced risk of *P. falciparum* clinical malaria, suggesting that by increasing the sensitivity of our clinical malaria case definition, IgG to these three antigens provide strong evidence of associations with protection against malaria. Previous studies carried out in the same area found that there is an association between the antibody responses to MSP3 and GLURP long synthetic peptides at the beginning of the malaria high transmission season and the reduced risk of clinical malaria [31], although two recent studies in Ghana and Senegal could not confirm those findings [14, 32]. The reasons for these discordant observations may be due to the variations in the technical procedures used for ELISA, the study design, the genetic background of study population as well as the pattern of malaria transmission. The major role may be played by malaria transmission level through the variations in the degree of exposure to particular antigens have probably the strongest influence on outcomes [21].

IgG1 responses to MSP3, GLURP R0 and GLURP R2 were associated with a reduced risk of malaria when the analysis is adjusted with age for all parasitemia thresholds defined. The same observation was seen for IgG3 to MSP3, and GLURP R2, with significant associations with IgG3 to MSP2b also observed for the >2500 and >5000 thresholds. These data suggest a role for cytophilic IgGs in protection against clinical malaria. IgG2 and IgG4 to MSP3 and GLURP R2 and IgG4 to MSP2b were also associated with a reduced risk of malaria. These data confirm those previously reported suggesting that IgG2, which preferentially binds the Fcγ receptor IIa 131H allele, is associated with protection against severe malaria [33–37]. However another study has shown that IgG2 is associated with susceptibility to malaria [38]. In contrast to four other antigens, no association was found for IgG subclass responses to MSP2a. It clearly appears that the pattern of the responses induced by this antigen is different from MSP3, MSP2b, GLURP R0 and GLURP R2. It is unknown whether the differences in MSP2a antibody reactivity observed in this study are related to its structural conformation with the tandem repeat sequences, or the polymorphic nature of some of these repeat sequences.

## Conclusion

On the basis of the data presented in this study, we conclude that natural acquired immunity to malaria antigens is related to age and transmission season levels in children less than 5 years. All the tested antigens are inducing antibody responses, which are associated with clinical malaria protection. Notwithstanding the factors that may affect the pattern of the immunological responses induced by these malaria vaccine candidates, the data presented in this paper are strongly in support of the inclusion of these antigens in malaria vaccine formulations.

## Abbreviations

*P. falciparum*: *Plasmodium falciparum*
MSP3: Merozoite Surface Protein 3
MSP2a: Merozoite Surface Protein 2a
MSP2b: Merozoite Surface Protein 2b
GLURP R0: Glutamate Rich Protein R0
GLURP R2: Glutamate Rich Protein R2
IgG: immunoglobulin G
IgG1: immunoglobulin G1
IgG2: immunoglobulin G2
IgG3: immunoglobulin G3
IgG4: immunoglobulin G4
SDH: Saponé Health District
RDT: rapid test diagnostic
WBC: white blood cells
SOP: standard operating procedure
AIA2: Afro Immuno Assay 2
OD: optical density
ELISA: enzyme-linked immunosorbent assay
CNRFP: Centre National de Recherche et de Formation sur le Paludisme
RT: room temperature
